# Description and Evaluation of a Visual Acuity Assessment Paradigm Based on Spatial Frequency-Modulated Form Discrimination

**DOI:** 10.1167/tvst.15.9.15

**Published:** 2026-09-23

**Authors:** Jonathan Lansey, Indrani Sirivella, Rahul Negi, Shaun R. Patel, Shrikant R. Bharadwaj

**Affiliations:** 1 REACT Neuro, Inc., Cambridge, MA, USA; 2 Prof. Brien Holden Eye Research Centre, L V Prasad Eye Institute, Hyderabad, India; 3 Centre for Technology Innovation, L V Prasad Eye Institute, Hyderabad, India; 4 Brien Holden Institute of Optometry and Vision Sciences, L V Prasad Eye Institute, Hyderabad, India

**Keywords:** blur, cognition, eye exam, higher-level visual processing, linguistics, spatial frequency discrimination

## Abstract

**Purpose:**

To describe and evaluate a visual acuity assessment paradigm based on spatial frequency-modulated form discrimination for induced and disease-driven vision loss.

**Methods:**

The Patterned-Optotype-in-Noise Task (POINT) consisted of arrow-shaped random noise patterns, each with a 1/f^2^ amplitude spectrum overlaid on a low-pass filtered background. Stimulus difficulty varied logarithmically across 22 levels by adjusting the background cutoff spatial frequency, relative to foreground. Monocular acuity was measured for POINT stimuli and letter optotypes in 20 adults (21–46 years) with 0–4D of induced blur and in 50 patients (9–77 years) with vision impairment from ocular pathologies/uncorrected refractive error.

**Results:**

The POINT demonstrated systematic loss in visual acuity with induced optical blur and eye pathologies, like the standard letter acuity test (*P* < 0.001). The losses with induced blur in the POINT followed standard Fourier optics predictions of spatial-frequency discrimination between the background and foreground pattern. The test-retest repeatability of POINT was also comparable to letter acuity testing (mean difference ± 95% limits of agreement: +0.04 units ± 0.2 units), but with limited agreement in the acuity measures obtained between the two tests.

**Conclusions:**

The POINT is a reliable technique for estimating visual acuity with induced and disease-driven vision loss. However, caution should be exercised when directly comparing these outcomes to standard letter acuity tests owing to fundamental differences in stimulus construction and task requirements.

**Translational Relevance:**

The POINT offers a physiologically and theoretically grounded complement to standard assessment of visual acuity in clinical, research and public eye health settings.

## Introduction

High-contrast visual acuity is by far the most important outcome measure in an eye examination. Fundamental definitions of vision impairment,^1^ thresholds for triage in mass eye screenings,^2^ practical applications like passing an eye examination for driving license^3^ and clinical trials evaluating the efficacy of new ophthalmic interventions all consider visual acuity as their fundamental outcome measure.^4^ The task typically involves presenting high contrast optotypes with broadband spatial frequency spectrum from a specific distance and determining the optotype with the smallest angular subtense that is correctly resolved by the individual.^5^ Screening tests for visual acuity use optotypes of specific angular subtense and triage individuals based on whether they are able/unable to resolve the details.^6^

The fundamental design of the eye chart used for assessing visual acuity has changed little since its origin in 1862.^7^^,^^8^ The changes primarily include regularizing the optotype shapes and their size progression, number and spacing per line.^9^^,^^10^ There are several challenges with the present design of visual acuity testing. One, the optotypes chosen for the test ought to be equally legible such that a certain level of vision degradation in the patient impairs the legibility of optotypes equally. Although this may be the case for standard Sloan optotypes, this is not a guarantee for charts developed in other languages.^10^^–^^12^ Second, the broadband optotypes used in standard acuity charts are assumed to be resolved using a fixed range of object frequencies (2.5 cycles/letter), independent of their size.^8^ However, empirical data shows the usage of lower object frequencies (∼1.3 cycles/letter) for resolving smaller optotypes compared to those used for resolving larger optotypes (∼2.5 cycles/letter). This lack of scale invariance complicates the interpretation of acuities measured across different levels of vision impairment.^13^ Third, the guessing rates during psychophysical testing vary across acuity charts. The Tumbling E or Landolt C charts typically show targets in only one of eight orientations, resulting in a 12.5% guessing rate of correct responses. In contrast, the guessing rate for Sloan optotypes is 10% if the individual is aware of which 10 letters in the English alphabet series are presented, or it will be only 3.8% if any of the 26 letters are expected to be presented. Guessing rates for eye charts created in other languages are likely to depend on how many optotypes from the local alphabet series are used for chart construction. Fourth, although visual acuity is primarily determined for foveal vision, shrinking optotypes may still be read by individuals with dysfunctional foveae using their peripheral vision (e.g., scotomas arising from scarred retina in age-related macular degeneration or microtropia).^14^^,^^15^ Similarly, the optotypes may show doubling or ghosting in patients with exaggerated optical aberrations (e.g., keratoconus or post-keratorefractive surgeries).^16^^,^^17^ This may lead correct identification of optotypes or missing optotypes that are not expected for their level of disease severity. Both issues may lead to misinterpretations about the overall visual health status of the patient.^18^ Fifth, visual acuity outcomes also depend on chart contrast and luminance polarity (black letters on white background or vice versa).^19^^–^^21^ Charts constructed with reverse polarity tend to show greater deterioration in acuity with contrast reduction and optical blur, relative to the former.^19^^–^^21^ Finally, the charts using specific language optotypes rely on the individual's basic literacy and cognitive level to comprehend these optotypes.^22^^,^^23^ These limitations may, however, be partially overcome by using charts that simply involve identifying the optotype orientation (e.g., the Landolt C charts) or those involving the identification of more generic shapes (e.g., Lea symbols, Kay pictures).^24^^–^^31^ Apart from the standard ones, other forms of visual acuity tests have also been developed but with one or more of the same features as the standard charts described above (e.g., vanishing optotypes^32^^,^^33^ and acuity charts created by Horiguchi et al.^34^ and Adhikari et al.^35^).

The present study introduces the Patterned-Optotype-in-Noise Task (POINT), a visual acuity test paradigm that is based on broadband spatial-frequency discrimination between a patterned foreground and a noisy background. This POINT may be executed in much the same way as standard acuity tests, even while its construction inherently overcomes some of the challenges associated with standard acuity charts (e.g., object spatial frequency dependence, linguistic dependence, luminance polarity dependence and localized form perception). The purpose of this study is to (1) describe the construction of the POINT stimuli, (2) determine changes in visual resolution with these stimuli for varying levels of induced optical blur and vision loss occurring from organic ocular pathologies, (3) compare the outcomes of POINT with the standard letter acuity measurements, and (4) report its test-retest variability, vis-à-vis, the letter acuity test. For the second and third aims, the study hypothesized that visual resolution with the POINT stimuli will monotonically deteriorate with increasing vision impairment and this deterioration will agree with the losses observed in the letter acuity test. For the last aim, the study hypothesized that the test-retest variability of the POINT will be like that of the standard letter acuity chart.

## Methods

### Stimulus Construction and Characteristics

**Figure 1.**
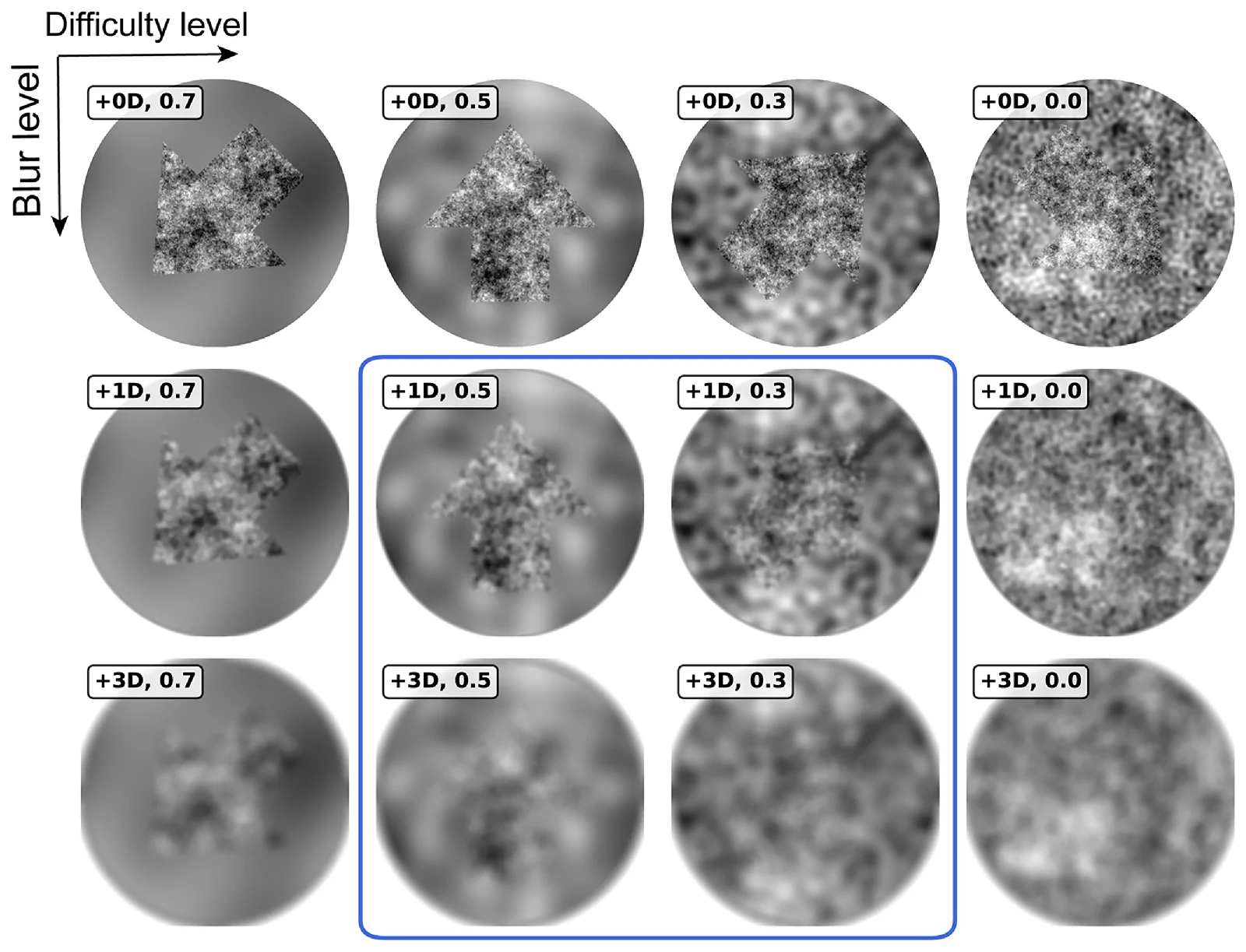
Representative POINT stimuli with increasing levels of difficulty used for the assessment of resolution acuity in this study. The cutoff frequencies of stimuli in columns 1–4 are 0.32, 0.76, 1.94, and 4.97 cycles/° (0.003, 0.007, 0.018, and 0.046 cycles/pixel on a 1080-pixel image), respectively. Blurred images are generated with a pillbox point spread function of radius 0° (*top row*), 0.11° (*middle row*), and 0.34° (bottom row), equivalent to 0D, 0.97D (rounded off to 1D), and 3.00D of defocus (0, 12, and 37 pixels, respectively). These simulations assume a pupil diameter of 4 mm, viewing distance of 2 m, and an image height of 35 cm. Images highlighted in the blue box are used to demonstrate the impact of optical blur on the POINT stimuli (see Fig. 2 for details). See also Figure A1 for the full set of POINT stimuli across all eight orientations used for visual acuity assessment.

Stimuli in POINT consisted of a target shape filled with random 2D noise having a 1/f^2^ spatial frequency power distribution across all orientations, where f is radial frequency in cycles per image width (Fig. 1). This pattern was generated by sampling a matrix of random phases uniformly distributed between 0 and 2*π*. Each phase and power value corresponded to a sinusoidal grating with spatial frequency equal to its distance from the center in the frequency domain. The background was a low-pass filtered version of the same noise pattern, achieved by applying a circular low-pass mask to the power spectrum. This low-pass spatial frequency filter removed energy in the background above a certain frequency threshold, henceforth referred to as the cutoff frequency (f_c_). For this instantiation of POINT, the stimuli were generated on a 1080 × 1080-pixel grid. The discrete Fourier transform of this grid yields a frequency-domain resolution of Δf = 1/1080 ≈ 0.001 cycles/pixel (0.1 cycles/° at 2 m viewing distance) (i.e., the coarsest nonzero frequency component corresponds to one cycle across all 1080 pixels). On the other end, the Nyquist limit is 0.5 cycles/pixel (54 cycles/°), corresponding to one cycle every two pixels. The transition around f_c_ was shaped by a raised-cosine filter with a gain function H(f): H(f) = 1 for f < f_c_ − 0.25, H(f) = 0.5 × [1 + cos(*π*(f − f_c_ + 0.25)/0.5)] for f_c_ − 0.25 ≤ f ≤ f_c_ + 0.25, and H(f) = 0 for f > f_c_ + 0.25, where, f is radial frequency in cycles per image width and the 0.5-cycle-wide transition band ensures a smooth roll-off rather than an abrupt cutoff which would have introduced high frequency artifacts. The final target and background were created by combining their respective phase and power information via an inverse discrete Fourier transform, then compositing them in the spatial domain using a mask of the target shape.

**Figure 2.**
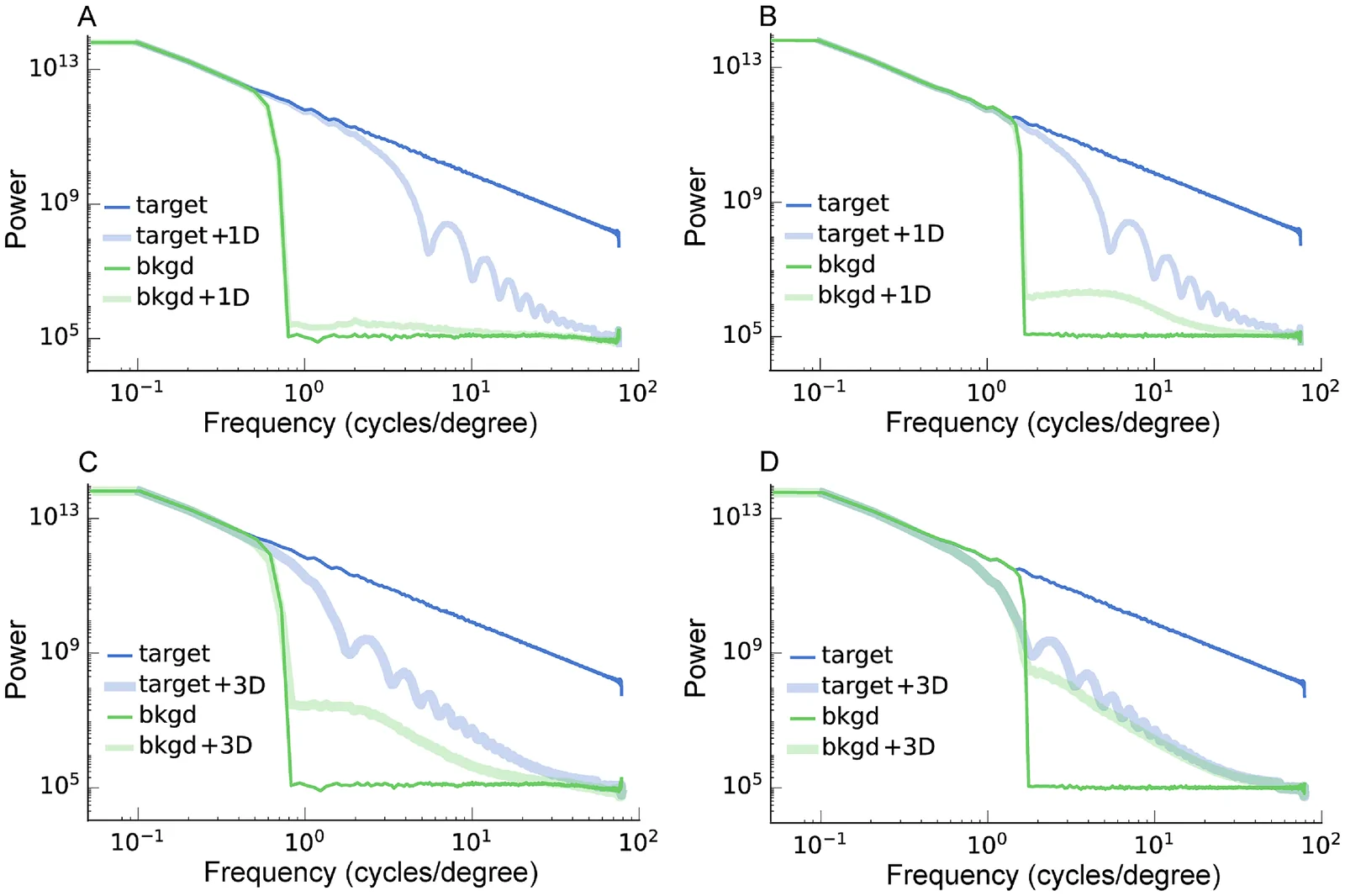
Radially-integrated 1/f^2^ power spectra of the foreground (target) and background (bkgd) corresponding to the stimulus panels highlighted by the blue bounding box in Figure 1 for +1D blur (**A** and **B**) and +3D blur (**C** and **D**). Panel A shows that there remains a significant difference in power spectrum between the foreground and background at +1D of blur for a target with relatively lower cutoff frequency (f_c_). With higher f_c_, the background and foreground power spectra come closer to each other (**B**). Panels **C** and **D** show the foreground and background spectra for the same target as panels **A** and **B**, but with +3D blur. With higher f_c_ and a higher level of blur, the power spectrum of the foreground and background overlap making target identification challenging. The non-zero power floor above f_c_ (∼10^5^) arises from eight-bit quantization of standard .png images: mapping continuous-valued pixel intensities to 256 discrete gray levels introduces rounding error that acts as low-level white noise uniformly distributed across all spatial frequencies (predicted floor: *N*^2^/12 ≈ 10^5.0^, where *N* = 1080 is the image dimension in pixels).

Low-pass filtering of the background creates a difference in the grain pattern, rendering the foreground target pattern visible relative to the background. Task difficulty is altered by manipulating the extent of low-pass filtering of the background noise while retaining the power distribution of the foreground; the larger the difference in the power distribution between the foreground and background, the easier it is to identify the pattern (Fig. 1). This is apparent from the power spectra of the target and background patterns of the four images shown in Figures 2A–D. The background image with the lowest value of f_c_ is the most heavily low-pass filtered image in the POINT series. Consequently, this image shows the largest difference in the power spectrum between the foreground and background and has the most prominent foreground pattern, relative to the background (Fig. 1, top row, left column). In contrast, the most difficult image in the POINT series has a background f_c_ closest to the cutoff value of the foreground. Consequently, this image has the least prominent foreground pattern, relative to the background (Fig. 1, top row, right column). Using the same logic, individuals who require a large difference in the spectral distributions between the foreground and background for pattern identification may be deemed to have poorer vision relative to those who can identify the foreground pattern with narrower separation in the foreground and background spectral distributions.

The target shape was an arrow icon modelled after the Unicode Right White Arrow (U+021E8, ⇨) (Fig. 1). Each image was 1080 pixels in resolution with a Michelson's contrast of 98.6% between the darkest and the brightest pixel in the image. The images were composed using a hard-edged binary mask in which the pixels inside the arrow defined the foreground pattern and those outside forming the background pattern. The entire stimulus set contained 22 logarithmically spaced levels of task difficulty (see, Appendix I for full set of POINT images), implemented by varying the low pass filter cutoff frequency of the background from 0.0009 cycles/pixel to 0.12 cycles/pixel (easiest to hardest in difficulty). The size and shape of the foreground arrow pattern remained fixed across difficulty levels. Eight variations of the arrow pattern were constructed for each difficulty level, with the arrows pointing in the four cardinal (0°, 90°, 180°, 270°) and four intercardinal directions (45°, 135°, 225°, 315°).

### Impact of Blur on POINT Stimuli

To determine the impact of optical blur on the POINT stimuli, the images of different difficulty levels shown in Figure 1, top row were blurred by convolving them with a circular (pillbox) point spread function. The PSF radii were chosen to approximate 1 D and 3 D of equivalent defocus using the relation |ΔD| ≈ (2/p) × (r · H)/(N · z), where p is pupil diameter (4 mm), r is the PSF radius in pixels, H is the displayed image height (0.35 m), N is the image resolution (1080 pixels), and z is the viewing distance (2 m). This yields radii of 12- and 37-pixels equivalent to 8.3 arcmin and 22.2 arcmin on the screen, respectively. The resulting diopters of blur are 0.97 D (rounded off to 1D) and 3.00 D. This approach using the pillbox filter directly models the finite aperture of a lens and has been widely used in depth-from-defocus literature to approximate a real optical blur filter.^36^ The resultant images are shown in Figure 1, middle and bottom rows. Their amplitude spectrum showed the expected low-pass filtered profiles, with greater impact of the blur on the amplitudes of the higher than lower spatial frequencies (Fig. 2). A small amount of optical blurring made the power spectra of the foreground and background indistinguishable for the images with a high background f_c_ value (i.e., “difficult” stimuli) (Fig. 1 right column, Figs. 2B, 2D), relative to those with low background  f_c_ value (i.e., the “easy” stimuli) (Fig. 1 left column, Figs. 2A, 2C). Visibility of the foreground arrow, relative to the background progressively declines with increasing background f_c_ value (Fig. 2, comparing the columns). Similarly, the difference in the spectral pattern shrinks between the foreground and background with an increase in the magnitude of optical blur (Fig. 2, comparing the rows). With a fixed level of blur, there is a specific stimulus image below which the target will be visible, and above which it will be indistinguishable from the background.

### Participants

All participants were recruited for this prospective study from the student, staff and patient pool of the L V Prasad Eye Institute (LVPEI), Hyderabad, India. The study adhered to the tenets of the Declaration of Helsinki, and it was approved by the Institutional Review Board of LVPEI, Hyderabad. Written consent form was obtained from all adult participants. For participants under 18 years age, written consent forms were taken from parent or legal guardian, and verbal assent was taken from the child. Visual acuities with POINT and standard letter acuity test were determined at baseline (i.e., no induced blur) and for a range of vision impairment by inducing 1–4 D of optical blur in otherwise visually-healthy participants (induced blur cohort). Twenty participants (13 males), aged 21–46 years of age, belonged to this cohort. All participants in the induced blur cohort had best corrected visual acuity of 20/20 or better (≤0 logMAR) in both eyes, with no history of ocular or neurological disease. Four of these participants were myopic (Mean ± 1 SD spherical equivalent refraction: −1.75 ± 0.79 D), two were hyperopic (mean ± 1 SD spherical equivalent refraction: +1.00 ± 0.00 D) whereas the remaining were emmetropic (spherical equivalent refraction within ± 0.5 D). Astigmatism was ≤ −1.00 D in all participants in this cohort. All participants were spectacle wearers. Data was also collected from 50 participants (33 males), aged 9–77 years, with a heterogeneity of ocular pathologies (disease cohort). These included glaucoma (*n* = 6), cataract (*n* = 6), retinal pathologies (*n* = 11), corneal pathologies (*n* = 12), amblyopia (*n* = 5), post-strabismus surgery (*n* = 1) and uncorrected refractive errors (*n* = 9). The refractive error in this cohort ranged from +6.50 D to −11.25 D spherical equivalent of refraction, with astigmatism ranging up to −5.00 D. Visual acuities of participants in this cohort were recording in their presenting condition, with (*n* = 16) or without (*n* = 28) their refractive correction. Visual acuities of the three moderate to high myopes were measured unaided. The vision loss of participants in this cohort was therefore due to a combination of their ocular pathology and uncorrected refractive error. Three participants in the disease cohort declined to participate after signing the informed consent form and were excluded from the study.

### Procedure

Monocular visual acuity was measured in all participants with their natural pupils and accommodative states in an otherwise darkened room. The order of the testing acuities using POINT and the letter acuity test was randomized across participants. One eye of the participant in the induced blur cohort was randomly chosen for induction of optical blur. The testing was performed with the participant's best refractive correction, if any, placed on a trial frame at a typical vertex distance of 14 mm. Spherical powered trial lenses of +1.0 D, +2.0 D, +3.0 D, and +4.0 D were then placed in the trial frame to induce vision impairment. The order of lenses used for testing was randomized across participants. No lenses were placed before the participant's eye in the baseline zero defocus condition. In the disease cohort, the eye with the manifest disease (in cases of unilateral affliction) or the eye with the more severe disease presentation (in cases of bilateral affliction) was chosen for testing. The fellow eye was occluded throughout the test in both cohorts.

The POINT stimuli and letter optotypes were displayed on the same luminance and gamma-calibrated CRT monitor (P1230 CRT Monitor, HP Inc.; display size of 45 × 35 cm with resolution of 1600 × 1200 pixels; 0.49 and 0.98 arcmin per pixel at 2 m and 1 m viewing distance; 85 Hz refresh rate). Both forms of visual acuity testing typically happened from a 2 m viewing distance, with +0.50 D correction for the optical vergence demand to simulate a standard 4 m working distance. The only exceptions to this were acuity testing using the POINT stimuli with +3D and +4 D induced blur, in which the testing happened from a 1 m viewing distance, with +1.0 D correction for the optical vergence to simulate 4 m working distance. Halving the distance to the stimulus results in a log(1/2) decrease in stimulus spatial frequency which was accounted for in our analysis and charting. The change in viewing distance was performed to ensure that the difficulty range of the POINT stimuli was within the range of deteriorated visibility with induced blur. The 0.5 D and 1 D working distance correction lenses were added to the same trial frame in which the refractive correction, if any, and the blurring lenses (for the induced blur cohort) were placed. The POINT stimuli subtended a visual angle of 10.0° and 19.9° at the viewing distances of 2 m and 1 m, respectively. The resulting spatial-frequency ranges corresponding to the stimulus background f_c_ were 0.14–17.6 cpd at 2 m and 0.07–8.8 cpd at 1 m.

A standard method of constant stimuli psychophysical paradigm was used to test visual resolution in both paradigms. Stimuli were presented in a randomized order across a fixed set of 11 difficulty levels based on pilot experiments of acuity loss for a given magnitude of vision impairment. Each target size was presented 10 times in random order, each for a duration of one second for POINT testing and 300-milliseconds for letter optotypes testing. Participants verbalized their responses at the end of each presentation (letters in the letter acuity test and arrow orientation for POINT stimuli). Pilot testing indicated that the 300 msec of target presentation used for letter acuity testing was not sufficient for participants with normal vision to correctly identify the arrow orientation of the easiest stimulus. The one-second presentation duration resulted in a more balanced distribution of correct and incorrect responses across stimulus difficulty levels that enabled a more reliable estimation of the threshold in participants. The POINT comprised of arrows in eight orientations, and it was an eight-alternate forced-choice task. The letter acuity test comprised of 10 standard Sloan optotypes. However, because the participants did not have knowledge of the optotypes that were presented, the task essentially became a 26-alternate forced choice task. No feedback was provided to the participant during either test paradigm. The entire testing paradigm was executed using custom-written software in the Psychtoolbox-3 interface of Matlab 2024a (Mathworks Inc, Natick, MA, USA).

In all ensuing analyses, visual acuity recorded using the letter acuity test are reported as the log_10_ of the minimum angle of resolution (logMAR) of the optotype stroke width needed for threshold level discrimination (i.e., 1/5th the angular subtense of the entire optotype). Similarly, acuities recorded using POINT are reported as the log_10_ of the minimum wavelength (where, wavelength = 1/f_c_) of the background needed for threshold level discrimination of the foreground pattern. These measures are comparable in that both describe the angular subtense (in arcmin) required for resolving the pattern. Psychometric functions were constructed for a plot of percentage correct response against target difficulty for each participant and each focus condition. Visual acuity thresholds were estimated by fitting the raw data to a logistic function (Equation 1). The lower asymptote of this function was fixed to the task's forced-choice chance rate (POINT: 1/8; letter optotypes: 1/26, noting that subjects were told the letters would be English and so they could have guessed any of the 26 letters in the alphabet not only the 10 Sloan Letters), and upper asymptote fixed at 1.0 (lapse rate was considered to be zero in this modeling, assuming no deviation in performance from attention loss, fatigue, etc). The threshold (location) x_0_ and slope k was optimized using the non-linear least squares method (SciPy curve fit, trust-region reflective), constraining x_0_ to the range of tested stimulus difficulty levels and k > 0. Initial values were set by the response midpoint for x_0_ and a moderate positive value for k. For the letter acuity test, x was the presented stroke width of the stimulus in log(arcmin); for POINT, x was the log of the low pass filter cutoff wavelength log_10_ 1/f_c_ adjusted for viewing distances of one or two meters depending on the viewing conditions. In both cases the horizontal center of the logistic curves were used for the final value.

(1) $$\begin{array}{c} p\;\;\left(correct\;|\;x\right)\;=\gamma +\left(1\;-\gamma \right)\;\left[1\;+\frac{1}{e^{-k\left(x-x_{0}\right)}}\right] \end{array}$$

The test-retest repeatability of POINT and the letter acuity test was determined on a subset of five participants each from the induced blur cohort and disease cohort based on their availability for repeated testing. The induced blur retest cohort was retested at 0 D, +2 D and +4 D of blur levels. The repeat measurement was obtained approximately five to six months after the first measurement. The experimental setup, test paradigm, and all other participant-related details remained identical between the two measurements.

## Results

**Figure 3.**
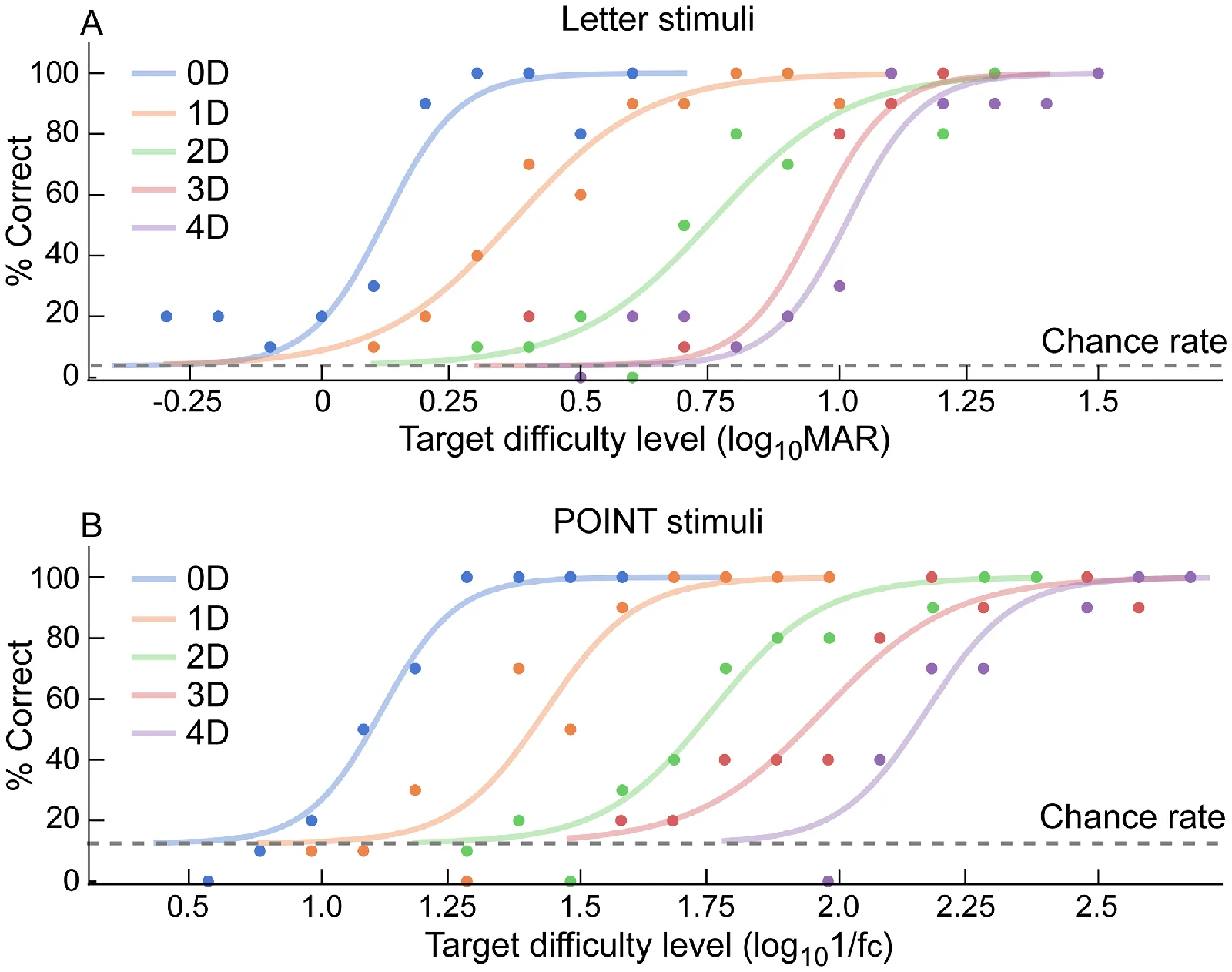
Representative psychometric functions of percentage correct identification of the targets plotted against the letter optotype size for different blur conditions in the letter acuity test (**A**) and the log_10_ of the cutoff wavelength (log_10_1/f_c_) of the background noise in the POINT (**B**) obtained from one participant in the induced blur cohort. The psychometric functions shifted to the right with increasing blur, indicating a systematic worsening of visual acuity with blur in both paradigms.

Visual acuity data was successfully collected from all study participants. Figure 3 shows representative psychometric functions for letter acuity test (Fig. 3A) and POINT (Fig. 3B) stimuli from one participant in the induced blur cohort. The typical sigmoid shaped psychometric functions and their corresponding thresholds systematically shifted to the right with an increase blur in both test paradigms (Fig. 3). These results are in line with the acuity losses described in the optical analysis shown in Figure 2. The delta shifts in the psychometric functions along the abscissa per dioptric increment of blur decreased in both tests, with this effect being more prominent in the letter acuity test than in the POINT test (Fig. 3). This indicated saturation of acuity estimates at larger blur levels, more so for letters than for the POINT stimuli.

**Figure 4.**
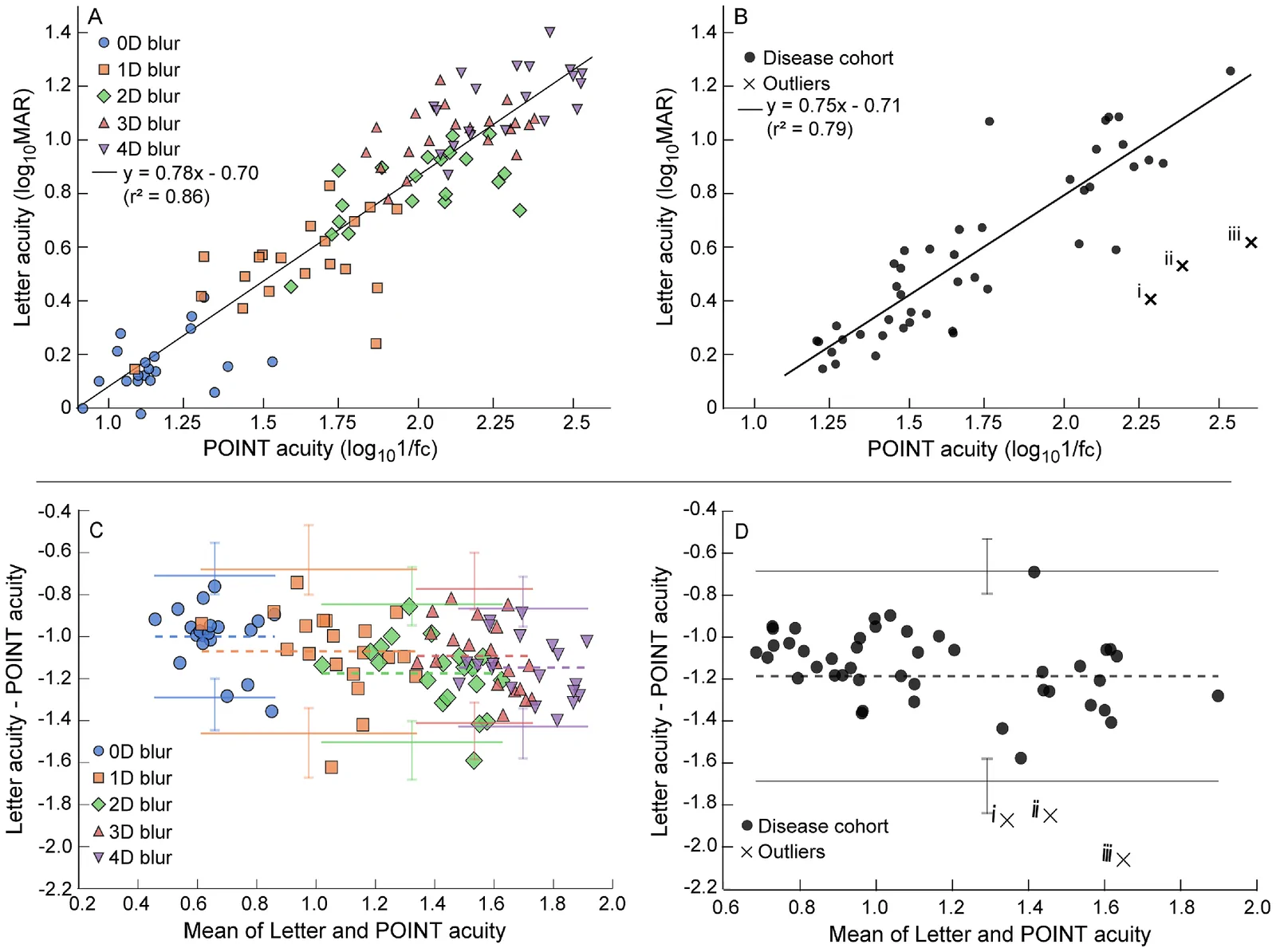
Visual acuity tested using the letter stimuli plotted against those obtained using POINT stimuli for the induced blur (**A**) and disease (**B**) cohorts. The blur levels are indicated using different symbols and colors in panel **A**. In each panel, the *solid line* is the best-fit linear regression equation describing the relation between the two tests. The three outliers in the disease cohort are identified with *crosses* in panel **B**. Panels **C** and **D** show Bland-Altman agreement plots of letter acuity versus POINT. The bias (*dashed line*) and limits of agreement [with 95% confidence intervals]^37^ are computed separately for each blur level in panel **C** and across the entire cohort in panel **D**.

The resolution thresholds obtained in both cohorts were normally distributed according to the Shapiro-Wilk test (W = 0.95, *P* = 0.33). Therefore parametric statistics were performed to analyze the data. Figure 4A plots the resolution thresholds obtained with the letter targets and POINT stimuli against each other for the different induced blur conditions. The mean (±1 SD) acuities for the letter targets with 0 D–4 D induced blur were 0.16 ± 0.11, 0.53 ± 0.17, 0.82 ± 0.14, 1.02 ± 0.10, and 1.14 ± 0.13 logMAR units, respectively. These mean values corresponded to delta changes of 3.34x, 1.54x, 1.24x, and 1.12x between adjacent values of blur increments. For POINT, the mean (±1 SD) acuities with 0 D–4 D induced blur were 1.16 ± 0.15, 1.60 ± 0.22, 2.00 ± 0.21, 2.11 ± 0.18, and 2.29 ± 0.17 log_10_ arcmin, respectively. These mean values corresponded to delta changes of 1.38x, 1.24x, 1.06x and 1.08x between adjacent values of blur increments. The losses in visual resolution between paradigms were well-correlated (Pearson's *r* = 0.93; *P* < 0.001), with a best-fit linear regression equation of y = 0.78x – 0.70 (*r*^2^ = 0.86). A two-factor repeated-measures analysis of variance (blur magnitude × testing paradigm) was performed to determine the statistical significance of the change in visual resolution with increasing blur magnitude in the two testing paradigms. The main effect of optical blur was statistically significant *F*(4, 76) = 442.94, *P* < 0.001] and Bonferroni-corrected pairwise comparisons among blur levels showed that every adjacent and non-adjacent pair differed significantly (all adjusted *P* < 0.001). The main effect of testing paradigm was also significant [*F*(1, 19) = 15.12, *P* = 0.001], and there was a significant interaction between blur magnitude and paradigm [*F*(4, 76) = 7.44, *P* < 0.001].

Data from the disease cohort was very similar to the induced blur cohort (Fig. 4B). Three participants with neuro-ophthalmic pathology (i: advanced optic disc damage; ii: ametropic amblyopia and iii: proliferative diabetic retinopathy) were identified as outliers in the disease cohort, with POINT measuring significantly worse visual acuity than the letter acuity test. The *Z*-score between their acuities were 3.05, 2.96, and 3.78, respectively, with these scores being computed as the standardized deviation of each participant's POINT to letter acuity difference from the cohort mean difference. After exclusion of these outliers, the two acuity measures were well-correlated with each other (*r* = 0.89; *P* < 0.001), with a best-fit linear regression equation of y = 0.75x – 0.71 (*r*^2^ = 0.79). The lesser than unity slope of the regression lines shown in Figures 4A and 4B indicated that the POINT stimuli became progressively harder to identify than the letter targets with increasing vision impairment either from induced blur or disease.

Bland-Altman analyses performed to determine the agreement in acuity levels obtained between the letter acuity test and POINT for both cohorts (Figs. 4C, 4D). The bias, 95% limits of agreement (LoA) along with their 95% confidence intervals are shown for each blur level in the induced blur (Fig. 4C) and disease (Fig. 4D) cohorts. For the induced blur cohort, biases ranged from −1.00 units for 0 D to −1.18 units for 2 D, with LoA widths between 0.56 and 0.78 units. For the disease cohort, the mean difference was −1.19 units with an LoA width between −1.69 and −0.69 units.

**Figure 5.**
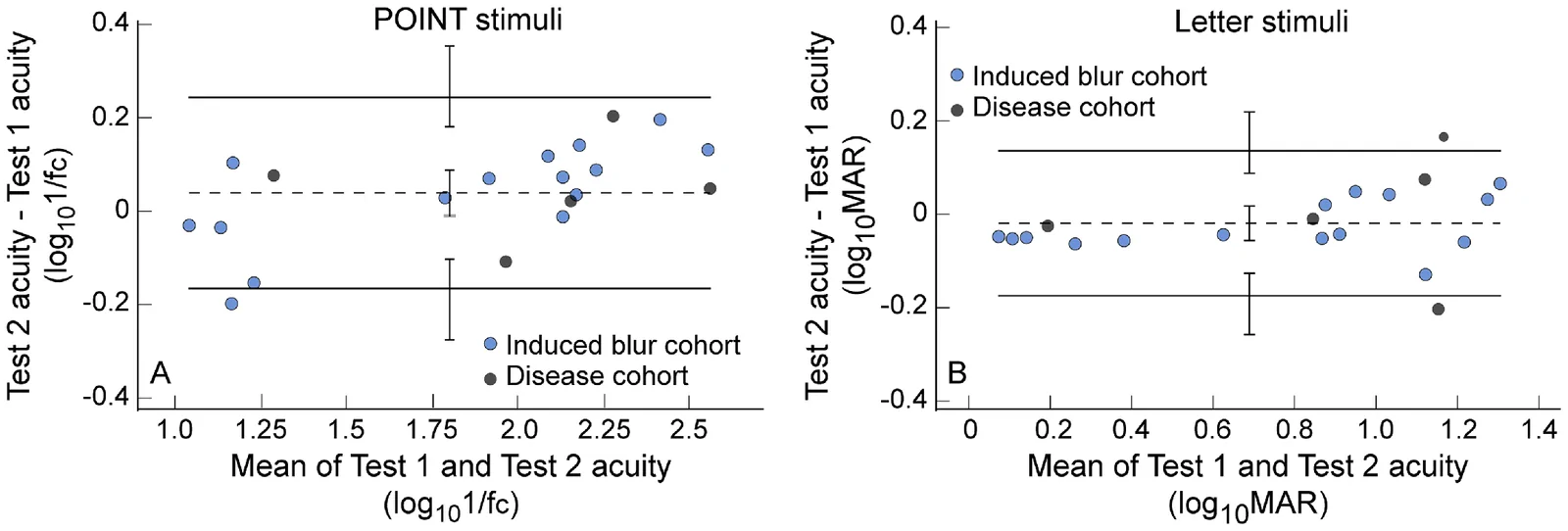
Bland-Altman plot of the difference between the test-retest results for the POINT test (**A**) and letter acuity test (**B**) plotted against the mean value of the two measurements in the respective tests.

The Bland-Altman analysis for test-retest repeatability for POINT stimuli showed a mean bias of +0.04 log_10_1/fc with the limits of agreement of −0.17 to +0.24 log_10_1/fc (Fig. 5A). For the letter acuity test, the mean bias was −0.03 log_10_MAR with the limits of agreement of −0.18 to +0.13 log_10_MAR for the same 10 subjects (Fig. 5B).

## Discussion

This study reports the construction of the POINT test for visual acuity assessment and compares the outcomes to standard letter acuity assessment. Certain features of the POINT stimuli are worth noting, relative to the targets used in standard acuity tests. First, the POINT stimuli are constructed on first principles of spatial frequency discrimination that is performed very early-on in visual processing (Fig. 1).^38^^,^^39^ The test involves discriminating the orientation of a familiar shape (arrows), the difficulty of which increases with the prominence in the spatial frequency structure of the background, relative to the foreground pattern. This is unlike standard eye charts that involve interpretation of complex-shaped patterns (especially, language optotypes) that may influence visual acuity outcomes from implicit biases arising from top-down knowledge of optotype familiarity.^40^ Second, the POINT stimuli are based on global form discrimination and not on optotype angular resolution per se. Visual acuity limits are assessed in the POINT test by retaining the target size and simply altering the spatial frequency content of the background patterns, relative to the foreground. Unlike standard optotypes,^13^ the fractal nature of 1/f^2^ power spectrum used to construct the POINT stimuli makes them inherently scale invariant (Figs. 1, 2).^41^ These may make the visual acuity assessment less complex to interpret in terms of retinal resolution across a range of vision impairments. Also, the acuity measurements become less vulnerable to inhomogeneities in resolving power of the eye across the visual field (e.g., those arising from scotomas due to retinal pathology) (Fig. 4). Third, since the POINT stimuli are constructed using standard grayscale luminance-based noise spectra (Fig. 2), the issue of luminance polarity affecting the visual acuity measurements in standard eye charts does not arise.^20^^,^^42^ Last, the POINT stimuli are agnostic to the linguistic capabilities of an individual, thus circumventing the need for different local language eye charts currently used in clinics across the globe.^12^ The present study results indicate that, much like the standard letter chart, visual acuities obtained using the POINT stimuli are sensitive to vision loss induced purposely with optical blur or those occurring naturally from organic pathologies of the eye (Figs. 4A, 4B). Also, like the standard letter chart, the test-retest repeatability of the POINT test showed limited bias, albeit with slightly larger limits of agreement for the latter than the former mode of acuity testing (Fig. 5). All these findings support the utility of POINT as a viable alternative to standard visual acuity assessments in clinical, research, and public eye health settings.

Even though the visual acuity outcomes between the POINT test and the standard letter acuity test were well-correlated in the two cohorts (Figs. 4A, 4B), the agreement between the two tests was relatively poor (Figs. 4C, 4D). This indicates that the outcomes of the two tests are not necessarily comparable, and any conversion from one form of acuity measurement to another must be made with caution. Should such a conversion be mandated, appropriate allowances are to be included in the converted results to account for this variability. This difference in acuity outcomes between the two tests is not necessarily surprising, given the fundamental differences involving stimulus construction and task requirement from the participant in the two tests. The present results also showed a saturating trend in the acuity estimates with increasing vision impairment, with the effect being more prominent for letter targets than for POINT stimuli (Fig. 3). This factor, combined with the lack of scale invariance in the letter targets,^13^ may be interpreted to indicate that larger sized letter targets are relatively easier to identify than comparable difficulty levels of the POINT stimuli (Figs. 4A, 4B). This may also contribute to the overall reduction in agreement between the two tests. The variability in the results may also arise from the relative unfamiliarity of participants to the POINT stimuli—participants in the two study cohorts are used to the routine form of acuity testing, but they may not be used to deciphering abstract shapes in a luminance noise field. The POINT stimuli were indeed shown for a longer time per presentation relative to the standard letter target (1-sec vs. 300-msec in the letter acuity test) in this study, potentially reflecting this difficulty/unfamiliarity. No explicit training was offered to the study participants in the POINT test, other than an overall orientation to the POINT test. The impact of familiarization with the POINT stimuli on task repeatability and on its agreement with standard letter tests is an issue for future exploration.

The present study has the following limitations. First, some of the aforesaid advantages of the POINT stimuli—specifically, their resilience to spatial inhomogeneities in ocular resolution, invariance to target luminance polarity and its applicability in patients across a range of cognitive capabilities—remain untested claims presently. These need to be tested explicitly in patients with such visual deficiencies in the future (e.g., patients with absolute scotomas arising from age-related macular degeneration or glaucoma^43^^,^^44^ and patients with deficient cognitive capabilities arising from neuro-developmental disorders).^45^ Second, the agreement between the POINT test and letter acuity was determined on a limited number (*n* = 70, across both cohorts) of conveniently sampled participants using psychophysical techniques typical of a laboratory experiment. The intended use of this test is in the clinic, in which some of the constraints imposed in the laboratory testing may not be possible (e.g., method of psychophysical testing, etc.). It remains unknown whether the results will continue to show similar trends under such testing conditions. The present study should therefore be considered as a proof of concept of a novel visual acuity testing paradigm based on spatial frequency discrimination, producing results comparable to standard letter acuity testing.

In conclusion, POINT measures acuity as structure-from-noise discrimination rather than letter/symbol identification, aligning with fundamental computations performed by the visual system. The POINT therefore offers a scalable, physiologically and theoretically grounded complement to standard acuity assessment in clinical, research and public eye health settings.

## Figures and Tables

**Figure A1.**
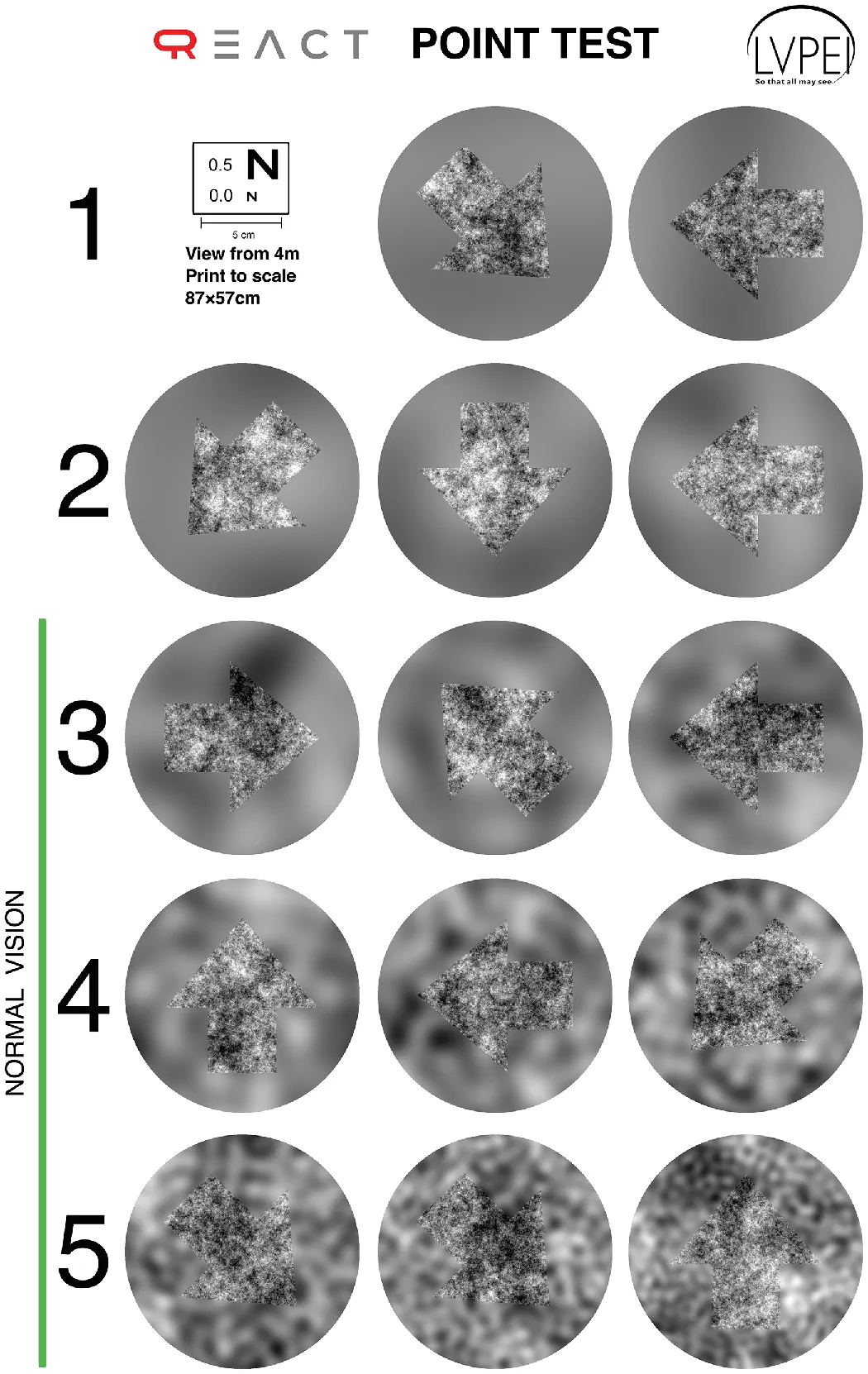
The POINT Stimuli Eye Chart That Could Be Used for Screening Visual Acuity.

## Appendix

### Appendix I.

The poster should be printed at 100% scale to 87 × 57 cm. Participants may be seated in front of the eye at a viewing distance of 4 m. They may then be asked to point towards the direction of the arrow with finger/hand gesture or verbally, starting with the easiest stimulus (1.0; corresponding to 20/200 acuity on a logMAR chart) and working the way downward until the arrow direction is no longer veridically identifiable. Visual acuity is determined by the most difficult circle that the participant was able to identify correctly.
